# Palliative Benefits of the Multimodality Approach in the Re-treatment of Recurrent Malignant Glioma: Two Case Reports

**DOI:** 10.4103/0973-1075.58464

**Published:** 2009

**Authors:** Arulponni TR, Janaki MG, Nirmala S

**Affiliations:** Department of Radiation Oncology, M.S. Ramaiah Medical Teaching Hospital, Bangalore - 560 054, India

**Keywords:** Recurrent malignant glioma, Resurgery, Reirradiation

## Abstract

Two young male patients treated seven and four years back, for malignant glioma, returned with recurrence at the same site, with a World Health Organization (WHO) Performance Score of four and two. Both underwent resurgery and received postoperative reirradiation of 5040 cGy in 28 fractions and concurrent Temozolomide 75 mg/m^2^ body surface area (BSA) daily, and one patient received additional adjuvant Temozolomide 250 mg (150 mg/m^2^ BSA). Both patients tolerated the treatment well with 16 and 14 months follow-up from the time of recurrence. They were symptom-free, with normal physical function and good mental state, and resumed their respective jobs.

## INTRODUCTION

This is a case report of two patients who presented with recurrent Anaplastic Oligodendroglioma Grade 3 and Anaplastic Astrocytoma (AA). Glioblastoma multiforme (GBM) and AA are the two most aggressive primary brain tumors with a grim prognosis despite advanced management. The median duration of patient survival is 12 and 24 months, respectively, with treatment.[1] The recurrence is inevitable; its management is often unclear and case-dependent. The neurological deficits such as motor dysfunction, seizures, visual deficits, and pressure effects, pose a dilemma in deciding the course of management. Recurrence occurring almost within the high-dose volume of postoperative Radiotherapy represents a major therapeutic challenge.[2] Reirradiation, however, was associated with acceptable toxicity when limited treatment volume and dose limits were respected and also depended upon the gap between the previous Radiotherapy and the recurrence.

## CASE REPORTS

### Case 1

**Figure 1:**
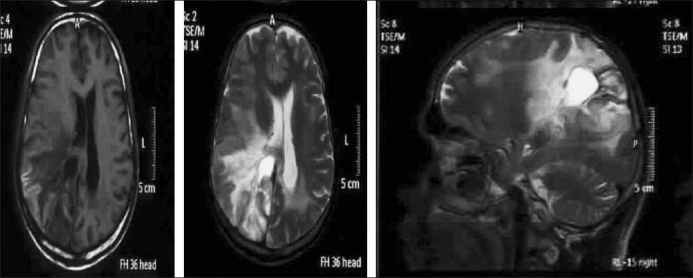
MRI of case one in the axial (T1 & T2 weighted) and sagittal views, demonstrating a mass lesion with extensive edema in the right parieto-occipital region with midline shift

Mr. N aged 35 years came to our Outpatient Department (OPD) on 4 July, 2008 with a history of (h/o) convulsions, left-sided weakness, swaying gait due to dragging of left foot, associated with headache since three months. He had no h/o vomiting. His WHO Performance score was four. He gave a h/o having undergone surgery seven years prior for convulsions and had received postoperative Radiotherapy and Chemotherapy for AA to the (R) parieto-occipital region. On examination (O/E) for the power on the left (L) side was grade 4/5, gait showed dragging of (L) foot and swaying to the left side. Computed tomography (CT) of the brain [Figure 1] showed SOL (space occupying lesion) in the right (R) parieto-occipital region. He underwent decompression of the lesion and histopathology showed Anaplastic Oligodendroglioma Grade III. The patient received postoperative radiation therapy (RT) of 3960 cGy and boost of 1080 cGy, 180 cGy/fraction, and concurrent Temozolomide 75 mg/m^2^ BSA given daily. After one week from the start of RT, the patient developed convulsions for which he was started on anticonvulsants. At one month follow-up he had persistent left-sided weakness, gait disturbance, and headache. He developed blurring of vision. His power was status quo and fundoscopy was normal. It was planned to start on adjuvant Temozolomide, but due to non-affordability and non-compliance he was lost to follow-up. On telephonic enquiry dated 31 July, 2009, the patient was fine, on Tab Gardenal 60 mg twice daily and he was seizure-free from seizure. He came for follow-up on 13 August, 2009. He is fine with no seizures, good mental status, normal gait, and no neurological deficits, so far, with a follow-up of 16 months from the time of recurrence.

### Case 2

Mr. B, an employee in a factory, age 23 years, received postoperative radiotherapy in 2004, for right parieto-temporal Astrocytoma. In September 2008, he underwent surgery for recurrence at the same site and his WHO performance score was 2 with repeated attacks of seizures. He was treated in a similar manner as the first patient. During the last two fractions of RT, he developed convulsions, which were uncontrollable with routine anti-convulsants. He was shifted to intensive care, intubated, and was on the ventilator for three days. The patient developed sepsis and was managed accordingly. He received adjuvant temozolomide of 250 mg, given for five days, once every 28 days, and completed six cycles. The patient is seizure-free from seizure, with a good physical and mental status, with a 14 month follow-up, and he has resumed his job.

In both the patients the details of the previous treatment were not available, but they have received postoperative radiotherapy for six weeks. Since the gap of the previous radiation was seven and four years, a total dose of reirradiation of 5040 cGy, in conventional fractionation, was decided upon, safely delivered, and tolerated well.

## DISCUSSION

Local recurrence of malignant glioma is a common problem in clinical practice. A standard management regimen for recurrence does not exist. The various options available are resurgery, radiotherapy, systemic therapy, and the best supportive care. However, the decision depends on the specific patient and tumor-related factors. Resurgery will relieve the mass effect as well as remove the hypoxic component of the tumor. Tumor debulking reduces the risk of death by 36%, which is statistically effective for survival.[3] The median duration of survival after resurgery is nine months with GBM and 22 months in AA and high quality survival is two-and-a-half months for GBM and 20 months for Anaplastic Astrocytoma.[4]

Patients with recurrent brain tumors have invariably undergone a full-course of external radiation previously making reirradiation more difficult and much more toxic. Various options are conventional radiotherapy, intensity modulated radiotherapy, temporary or permanent brachytherapy implants and single or multifraction stereotactic radiosurgery.[5] It is possible to go up to a dose of 30-40 Gy without any unacceptable clinical neurotoxicity under the following conditions: Good postoperative WHO performance status of 0-1, at least a one-year, disease-free interval, an initial WHO grade of 2 or 3, with a maximal tumor diameter of 3 cms.[3] The only concern is to achieve an optimal efficacy/toxicity ratio. In 97% of the cases, tumor recurrence is completely situated within the original 90% isodose line.[2] Hypofractionated stereotactic radiotherapy with a moderate total dose of no more than 30 Gy is safe, with a median overall survival of 9.3-15.4 months for AA and 7.9 months for GBM, suggesting a therapeutic benefit in the selected patient groups.[6]

Some patients will probably benefit from chemotherapeutic treatment, but there is still a debate over the most appropriate salvage agent. Different chemotherapy schedules such as procarbazine, irinotecan, and temozolomide are proposed. A randomized phase II trial demonstrates that temozolomide provides longer progression-free survival and better quality of life than the standard dose of procarbazine, in patients with recurrent GBM.[7] As the benefits of chemotherapy for AA and GBM are small, participation in clinical trials is appropriate. Implantation of bischloroethylnitrosourea (BCNU) polymers during resurgery is supported by a randomized trial shown to be superior to reirradiation alone.[8]

Meaningful palliation is possible for patients with recurrent malignant glioma using multimodality treatment. Long-term, disease-free survival occurs in fewer than 10% of the patients.[7] Median survival exceeding one year is expected, the main end-point being palliation of symptoms and good quality of life, which has been achieved in both our patients.
